# Abacavir, efavirenz, didanosine, with or without hydroxyurea, in HIV-infected adults failing initial nucleoside/protease inhibitor-containing regimens

**DOI:** 10.1186/1471-2334-5-23

**Published:** 2005-04-08

**Authors:** Susan Swindells, Calvin J Cohen, Daniel S Berger, Karen T Tashima, Qiming Liao, Bonnie F Pobiner, Jerry W Snidow, Gary E Pakes, Jaime E Hernandez, the NZTA4008 Study Team

**Affiliations:** 1 HIV Clinic, University of Nebraska Medical Center, Omaha, NE, USA; 2 Community Research Initiative of New England, Boston, MA, USA; 3 North Star Medical Center, Chicago, IL, USA; 4 Infectious Diseases, Miriam Hospital, Providence, RI, USA; 5 GlaxoSmithKline, Research Triangle Park, NC, USA

## Abstract

**Background:**

Hydroxyurea (HU) is an immunomodulatory agent that has been documented to enhance the antiretroviral activity of nucleoside reverse transcriptase inhibitors, such as abacavir (ABC) and didanosine (ddI), and would be expected to improve virologic efficacy.

**Methods:**

A 48-week, phase IV, multicenter, open-label, proof-of-concept clinical trial was conducted to evaluate second-line, protease inhibitor (PI)-sparing therapy with ABC/efavirenz (EFV)/ddI plus HU or without HU in HIV-infected subjects failing to achieve HIV-1 RNA ≤ 400 copies/mL after ≥ 16 weeks of treatment with lamivudine/zidovudine or lamivudine/stavudine, plus 1 or 2 PIs. Subjects were assigned to ABC (300 mg twice daily)/ EFV (600 mg once daily)/ ddI (400 mg once daily) plus HU (500 mg twice daily) (n = 30) or this regimen without HU (n = 24).

**Results:**

Baseline mean HIV-1 RNA was 3.86 log_10 _copies/mL and CD4+ cell count was 345 cells/mm^3^. A similar percentage of subjects in the non-HU arm (58%) and HU arm (53%) completed the study. Intent-to-treat: missing = failure analysis showed no differences in proportions of subjects in the non-HU and HU arms achieving undetectable plasma HIV-1 RNA levels at week 24 (<400 copies/mL: 58% [14/24] vs 57% [17/30], *P *= 0.899; <50 copies/mL (50% [12/24] vs 47% [14/30], *P *= 0.780). Median change from baseline in CD4+ cell count in the non-HU and HU arms at week 48 was +114 cells/mm^3 ^and -63 cells/mm^3 ^(*P *= 0.007), respectively. Both regimens were generally well tolerated, although more subjects in the HU arm withdrew prematurely from the study due to adverse events (23% vs 4%). Four cases of possible ABC-related hypersensitivity were observed.

**Conclusion:**

ABC/EFV/ddI was an effective and well-tolerated second-line regimen for nucleoside/PI-experienced HIV-infected subjects. The addition of HU blunted the CD4+ cell response, did not appear to enhance antiviral activity, and resulted in more treatment-limiting adverse events.

## Background

Combination antiretroviral therapy containing protease inhibitors (PIs) has contributed substantially towards delaying progression of HIV infection and decreasing morbidity and mortality [1,2]. However, PI-based regimens require strict adherence to ensure efficacy, and many of these regimens incur a high pill burden, complex dosing schedules, numerous drug interactions, and metabolic complications including lipodystrophy, hyperlipidemia, and elevated glucose levels. A high rate of virologic failure has been observed with PI-containing regimens in clinical practice [3]. In view of this, PI-sparing regimens using nucleoside reverse transcriptase inhibitors (NRTIs) and non-nucleoside reverse transcriptase inhibitors (NNRTIs) are attractive for initial and rescue use.

Studies in which the NRTI abacavir (ABC) was substituted for a PI have shown maintenance of virologic suppression and CD4+ cell elevation, with reduction in plasma lipid levels [4-7]. Substitution of the once-daily NNRTI efavirenz (EFV) for PIs has also allowed simplification of therapy with maintenance of virologic suppression [8]. Hirschel et al [9] have shown that when patients are switched from PIs to EFV they experience less virologic failure over a 1-year follow-up period than non-switchers. These results support the use of ABC and EFV in PI-sparing regimens.

Hydroxyurea (HU) is an immunomodulatory agent that depletes intracellular deoxynucleotides by inhibiting ribonucleotide reductase, thereby enhancing the antiretroviral activity of NRTIs and possibly accelerating intracellular phosphorylation [10,11]. Synergy has been shown *in vitro *between HU and didanosine (ddI) [10], and *in vivo *(murine AIDS model) between HU and ABC [12]. Regimens employing HU 500 to 1000 mg daily in combination with ddI have been reported to produce marked virologic suppression for up to 2 years [13-23]. Combinations of HU with ABC or EFV have also shown promise as treatment options for antiretroviral-experienced subjects [23,24]. However, to date, limited data have been presented on the therapeutic outcome in HIV-infected subjects treated with both EFV and ABC in HU-containing regimens [24]. The objective of this proof-of-concept study (NZTA4008) was to evaluate the long-term efficacy, safety, and tolerability of second-line therapy with ABC, EFV, ddI, with or without HU, in subjects who had failed their initial antiretroviral regimens.

## Methods

### Subjects

Male or female subjects 13 years of age or older were eligible for enrollment in the study if they had a screening plasma HIV-1 RNA value between 400 and 100,000 copies/mL, a CD4+ cell count ≥ 100 cells/mm^3^, had experienced virologic failure (HIV-1 RNA ≥ 400 copies/mL) after at least 16 weeks of initial antiretroviral therapy with 1 or 2 PIs with lamivudine and either zidovudine or stavudine, and had not previously received treatment with non-nucleoside reverse transcriptase inhibitors (NNRTIs), ABC, or ddI. Subjects were eligible if they had a hemoglobin level >9.0 g/dL (for women) or >10.0 g/dL (for men), a neutrophil count >1,000 cells/mm^3^, a platelet count >75,000 cells/mm^3^, an estimated creatinine clearance >50 mL/min, a serum lipase < the upper limit of normal, a serum pancreatic amylase level <1.5 times the upper limit of normal, and levels of hepatic aminotransferases < 5 times the upper limit of normal within 2 weeks prior to the baseline visit. Subjects were not eligible for enrollment if they had been on non-suppressive initial antiretroviral therapy for >1.5 years, were pregnant or breastfeeding, were receiving immunomodulating agents, an immunotherapeutic vaccine, or cytotoxic chemotherapeutic agents within 8 weeks before study start, had a history of pancreatitis or peripheral neuropathy within 2 months before study start or had been diagnosed with acute hepatitis within 6 months before study start.

### Study design

This was a Phase IV, randomized, open-label, proof-of-concept clinical trial that was conducted at 17 study sites in the United States between September 2, 1999 and April 27, 2001. The study protocol was approved by the institutional review boards at each study site, and all subjects provided written informed consent prior to their participation. Subjects were randomly assigned to receive one of two treatment regimens: ABC 300 mg twice daily, EFV 600 mg once daily, and ddI 400 mg once daily (the non-HU arm); or the ABC/EFV/ddI regimen with HU 500 mg twice daily (the HU arm). This study was planned for 48 weeks, but it concluded after the last subject completed 24 weeks of treatment because accrual was very slow due to the highly selective population sought. In the HU treatment arm, subjects were randomly assigned to start HU either at baseline or at week 8 in order to investigate whether delaying HU could prevent cytopenia and a decrease in CD4+ count.

ABC was supplied as 300-mg tablets of Ziagen^® ^(Glaxo Wellcome, Research Triangle Park, NC), EFV as 200-mg capsules of Sustiva^® ^(DuPont Pharmaceuticals, Wilmington, Delaware), ddI as non-enterically-coated 100-mg, 150-mg, and 200-mg tablets of Videx^® ^(Bristol-Myers Squibb, Princeton, New Jersey), and HU as 200-mg, 300-mg, and 500-mg capsules of Droxia^® ^(Bristol-Myers Squibb, Princeton, New Jersey). Doses of ddI were administered at least 30 minutes before a meal or 2 hours after a meal or snack. The once-daily dose of EFV was administered either in the morning or evening, with or without food. ABC and HU did not have specific dosing requirements regarding timing of doses with respect to meals.

### Study procedures

Subjects were evaluated at screening, baseline (day 1), and at weeks 4, 8, 16, 24, 32, 36, 40, and 48. Subjects were evaluated upon premature discontinuation of the study and at 4 weeks post-study discontinuation. For subjects randomized to receive HU beginning at week 8, an additional study visit was scheduled at week 12 to perform safety evaluations. Plasma samples were collected at the study visits for virology, immunology, hematology, and clinical chemistry assessments that were performed by a central laboratory (Consolidated Laboratory Services, Van Nuys, California). Plasma HIV-1 RNA levels were assessed in blood samples at screening and at all study visits using both the Roche AMPLICOR PCR Standard 1.0 assay (lower limit of quantitation [LLOQ] 400 copies/mL) and the Roche PCR assay Amplicor HIV-1 MONITOR UltraSensitive Version 1.0 (LLOQ 50 copies/mL) (both assays from Roche Diagnostics, Branchburg, New Jersey). CD4+ cell counts were determined by flow cytometry. Urinalysis was performed at baseline and at weeks 4, 12, 16, 24, and 48.

Clinical adverse events and laboratory abnormalities were assessed and graded according to the standardized AIDS Clinical Trials Group (ACTG) toxicity grading scales wherever possible (grade 1 or mild to grade 4 or severe). In cases of suspected HU-related Grade 2 or higher hematologic toxicities, hyperamylasemia, or neuropathy, HU was discontinued until the toxicity returned to equal to or less than Grade 2. HU was restarted at a reduced dose and could be subsequently increased to full dose. If amylase levels did not decrease following interruption of ddI, all study medications were to be interrupted. Persistent, recurrent, or Grade 3 or higher hematologic toxicities, hyperamylasemia, or neuropathy required permanent discontinuation of HU. Doses of ddI could be replaced with an alternate NRTI. If pancreatitis was diagnosed, all study drugs were to be permanently discontinued and the subject discontinued from the study, with or without elevated amylase levels. If a subject presented with symptoms consistent with a possible ABC-related hypersensitivity reaction, including fever, skin rash, fatigue, and gastrointestinal symptoms such as nausea, vomiting, diarrhea, or abdominal pain, therapy with ABC was permanently discontinued.

### Study endpoints

The primary efficacy endpoint was the proportion of subjects achieving plasma HIV-1 RNA levels <400 copies/mL at weeks 24 and 48. Secondary efficacy endpoints assessed at weeks 24 and 48 included the proportion of subjects achieving plasma HIV-1 RNA <50 copies/mL, change from baseline in HIV-1 RNA and CD4+ cell count, and proportion of subjects with at least a 50-cell increase in CD4+ cell count. During the 48-week study, assessments were made of the time to virologic failure and time to treatment failure. Virologic failure was defined by any of the following: HIV-1 RNA >400 copies/mL by week 24 of randomized treatment or repeated detection (>400 copies/mL) after initial suppression to undetectable levels (<400 copies/mL) or a 3-fold or greater increase in plasma HIV-1 RNA level from the nadir at week 8 or later not attributable to intercurrent infection or vaccination. Treatment failure was defined by one or more of the following: virologic failure, toxicity or other treatment-related withdrawal, or clinical disease progression (from CDC Categories A or B to Category C or death, or progression from Category C to death).

### Statistical analysis

Subjects were randomly allocated to the HU and non-HU arms. Within the HU arm, subjects were randomized either to treatment with HU starting at study baseline or to HU starting at 8 weeks post-baseline. Randomization was stratified by screening HIV-1 RNA (<10,000 copies/mL and ≥ 10,000 copies/mL). The total number of subjects originally planned for this study was 150 (80 in the HU arm [40 starting HU at baseline and 40 at 8 weeks]) and 70 in the non-HU arm. However, very slow enrollment necessitated participation by fewer subjects. The proportions of subjects achieving HIV-1 RNA <400 copies/mL and <50 copies/mL at week 24 was calculated using an intent-to-treat: missing = failure (ITT: M=F) analysis. This analysis, which included all randomized subjects, regarded as a treatment failure any subject with missing values or who did not initiate treatment, changed treatment, or prematurely discontinued randomized treatment for any reason. The ITT: M=F analysis was not performed at week 48 because of potential bias due to early termination of the study after the last subject completed 24 weeks. An ITT: observed analysis was performed at both week 24 and week 48, which included all data from subjects seen at each specific visit. Comparisons of proportions were made using the Cochran Mantel Haenzel test. Change from baseline and average area under the curve minus baseline (AAUCMB) in HIV-1 RNA (log_10 _copies/mL) and change from baseline CD4+ cell count were analyzed using the two-sample t-test and Wilcoxon sum rank test, respectively. Time to virologic failure and time to treatment failure were analyzed using the Kaplan-Meier method and compared between the treatment arms using the log-rank test. The study was not powered to show statistical differences between the treatment arms. Differences were deemed statistically significant if the *P-*value was <0.05.

## Results

### Baseline characteristics and subject disposition

Baseline demographic and disease characteristics were similar between the treatment arms (Table 1). Most of the subjects (≥ 87%) were male, and approximately one-half were Caucasian. Baseline median HIV-1 RNA in the non-HU (n = 24) and HU arms (n = 30) was 3.93 and 3.90 log_10 _copies/mL, respectively, and median CD4+ cell counts were 291 and 326 cells/mm^3^, respectively. A higher percentage classified CDC Category B was included in the non-HU arm (42% vs 17%), and a higher percentage classified CDC Category A in the HU arm (63% vs 38%); one-fifth of the subjects in each treatment arm were Category C. Prior to the study, the most frequently used NRTIs in each arm had been lamivudine, d4T, and the lamivudine 150 mg/zidovudine 300 mg combination tablet (Combivir^®^, GlaxoSmithKline, Research Triangle Park, North Carolina), and the most frequently used PIs had been nelfinavir mesylate and indinavir sulfate.

**Table 1:** Baseline demographics and disease characteristics

Characteristic	ABC/EFV/ddI (*N *= 24)	ABC/EFV/ddI/HU (*N *= 30)
Age, years		
Mean ± *SD*	39.5 ± 7.8	38.1 ± 8.4
Median (Range)	37 (29–62)	37 (26–59)
Gender, *n *(%)		
Male	21 (88)	26 (87)
Female	3 (13)	4 (13)
Race, *n *(%)		
Caucasian	13 (54)	16 (53)
African American	8 (33)	4 (13)
Hispanic	2 (8)	7 (23)
Other	1 (4)	3 (10)
CDC classification, *n *(%)		
Category A	9 (38)	19 (63)
Category B	10 (42)	5 (17)
Category C	5 (21)	6 (20)
HIV-1 RNA, log_10 _copies/mL		
Mean ± *SD*	3.86 ± 0.55	3.86 ± 0.67
Median (Range)	3.93 (2.86–4.73)	3.90 (2.73–4.82)
CD4 cell count, cells/mm^3^		
Mean ± *SD*	345 ± 192	346 ± 167
Median (Range)	291 (67–805)	326 (53–794)
Prior antiretroviral treatment, *n *(%)		
NRTIs	24 (100)	29 (97)
Lamivudine	16 (67)	19 (63)
Stavudine	11 (46)	18 (60)
Lamivudine/zidovudine combination tablet	10 (42)	10 (33)
Zidovudine	5 (21)	4 (13)
Zalcitabine	0	1 (3)
PIs	22 (92)	26 (87)
Nelfinavir mesylate	10 (42)	17 (57)
Indinavir sulfate	7 (29)	7 (23)
Saquinavir	3 (13)	2 (7)
Ritonavir	1 (4)	2 (7)
Lopinavir + ritonavir	1 (4)	1 (3)
Amprenavir	1 (4)	0
Premature withdrawal from study, *n *(%)	10 (42)	14 (47)
Adverse event^a^	1 (4)	7 (23)
Consent withdrawn	2 (8)	0
Protocol-defined virologic failure	5 (21)	1 (3)
Lost to follow-up	1 (4)	3 (10)
Protocol violation	0	1 (3)
Other	1 (4)	2 (7)

*Note: *ABC, abacavir; ddI, didanosine; EFV, efavirenz; HU, hydroxyurea; NRTIs, nucleoside reverse transcriptase inhibitors; PIs, protease inhibitors; SD, standard deviation.

^a^Adverse events that led to premature study withdrawal in the HU arm were diarrhea, dizziness, headaches, vomiting, rash on chest, insomnia (1 patient); pancreatitis (1); decrease in concentration, exacerbation of depression, nightmares (1); fatigue (1); flushing, fatigue, vomiting, nausea, palpitations (1); dizziness, incoherence (1); and possible ABC-related hypersensitivity reaction (1). The one patient in the non-HU arm who withdrew prematurely from the study did so because of fatal asphyxia, which was not considered related to drug treatment.

Of the subjects in the HU arm, 17 started HU at baseline and 13 at week 8. A similar percentage of subjects in the non-HU arm (58% [14/24]) and HU arm (53% [16/30]) completed the study. The reasons for premature withdrawal from the study are given in Table 1. A greater proportion of subjects in the HU arm withdrew prematurely due to adverse events (23% vs 4%), whereas a greater proportion in the non-HU arm withdrew due to protocol-defined virologic failure.

### Virologic response

**Figure 1:**
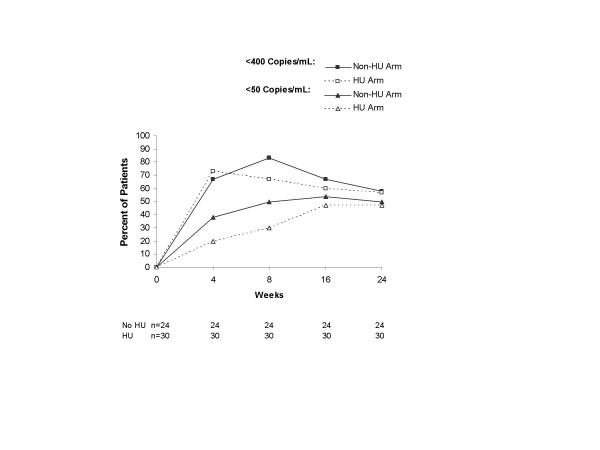
Proportion of subjects in the non-HU and HU arms who achieved plasma HIV-1 RNA <400 copies/mL and <50 copies/mL in the intent-to-treat: missing = failure analyses. HU = hydroxyurea.

**Figure 2:**
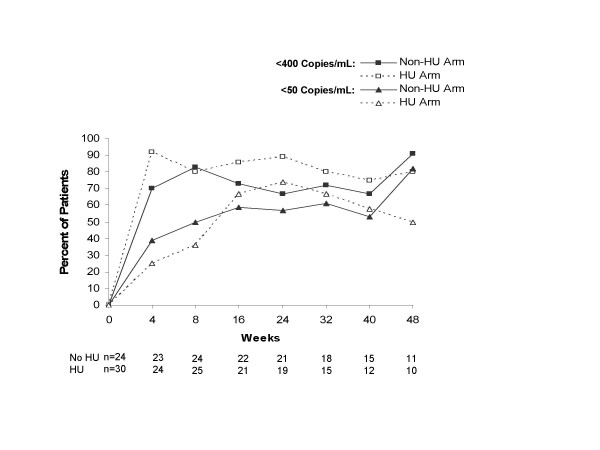
Proportion of subjects in the non-HU and HU arms who achieved plasma HIV-1 RNA <400 copies/mL and <50 copies/mL in the intent-to-treat: observed (**B**) analyses. HU = hydroxyurea.

At week 24, the proportion of subjects in the non-HU and HU treatment arms who achieved HIV-1 RNA <400 copies/mL was not significantly different, according to the ITT: M=F analysis (58% [14/24] and 57% [17/30], respectively; *P *= 0.899, Fig. 1) and ITT: observed analysis (67% [14/21] and 89% [17/19], respectively; *P *= 0.081, Fig. 2). Differences also were not observed at week 48, although the proportion of subjects with HIV-1 RNA <400 copies/mL tended to be higher in the non-HU arm in the ITT: observed analysis (91% [10/11] and 80% [8/10], respectively; *P *= 0.512).

Results with the 50-copy/mL assay paralleled those with the 400-copy/mL assay. At week 24, the proportion of subjects in the non-HU and HU treatment arms who achieved HIV-1 RNA <50 copies/mL was not significantly different in the ITT: M=F analysis (50% [12/24] and 47% [14/30], respectively; *P *= 0.780, Fig. 1) or the ITT: observed analysis (57% [12/21] and 74% [14/19], respectively; *P *= 0.301, Fig. 2). Differences also were not observed at week 48, although the proportion of subjects with HIV-1 RNA <50 copies/mL tended to be higher in the non-HU arm in the ITT: observed analysis (82% [9/11] and 50% [5/10], respectively; *P *= 0.137). No significant differences between the treatment regimens were observed in proportions of subjects achieving HIV-1 RNA <400 and <50 copies/mL in the protocol-specified subgroups of subjects with baseline HIV-1 RNA >10,000 copies/mL or between 400–10,000 copies/mL (data not shown). Median decrease in HIV-1 RNA from baseline tended to be greater in the HU arm than the non-HU arm at week 24 (-2.10 vs -1.45 log_10 _copies/mL, *P *= 0.070), but not week 48 (-2.05 vs -2.12 log_10 _copies/mL, *P *= 0.453). Median AAUCMB in HIV-1 RNA in the HU and non-HU arms was not different at week 24 (-1.57 vs -1.38 log_10 _copies/mL, *P *= 0.571) or week 48 (-1.66 vs -1.41 log_10 _copies/mL, *P *= 0.585).

**Figure 3:**
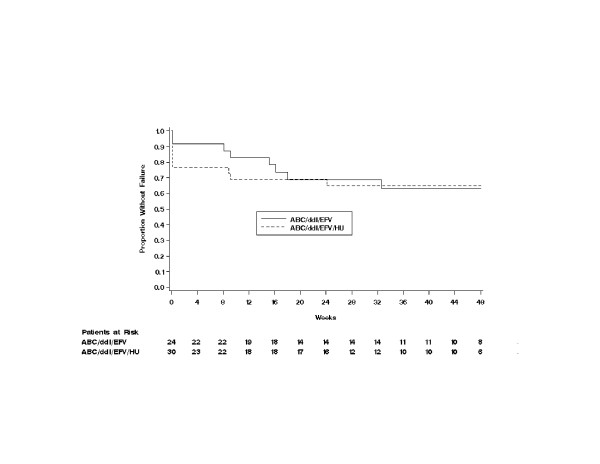
Time to virologic failure. ABC = abacavir; ddI = didanosine; EFV = efavirenz; HU = hydroxyurea. Virologic failure was defined by any of the following: HIV-1 RNA >400 copies/mL by week 24, repeated detection (>400 copies/mL) after initial suppression to undetectable levels (<400 copies/mL) or a 3-fold or greater increase in plasma HIV-1 RNA level from the nadir at week 8 or later.

**Figure 4:**
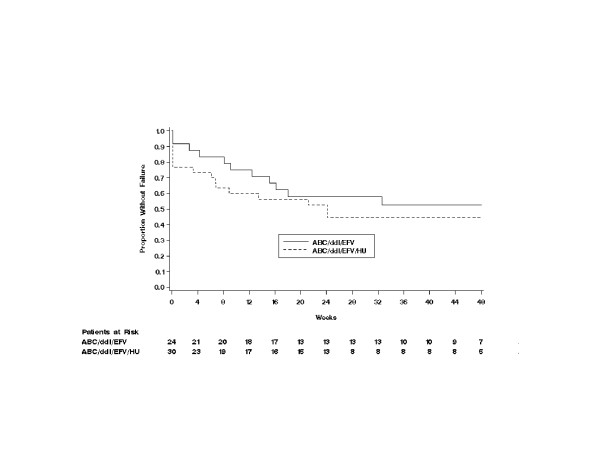
Time to treatment failure. ABC = abacavir; ddI = didanosine; EFV = efavirenz; HU = hydroxyurea. Virologic failure was defined by any of the following: HIV-1 RNA >400 copies/mL by week 24, repeated detection (>400 copies/mL) after initial suppression to undetectable levels (<400 copies/mL) or a 3-fold or greater increase in plasma HIV-1 RNA level from the nadir at week 8 or later.

The time to virologic failure did not differ between the non-HU arm and HU arm (*P *= 0.808, Fig. 3). In several subjects, a time to virologic failure of 0 was observed due to their lack of virologic response (continued or dropped out) during the first 24 weeks. No statistically significant difference was noted in the time to treatment failure between the two treatment arms (*P *= 0.418), although the non-HU arm demonstrated slightly longer survival time to treatment failure than the HU arm (Fig. 4).

### Immunologic response

**Figure 5:**
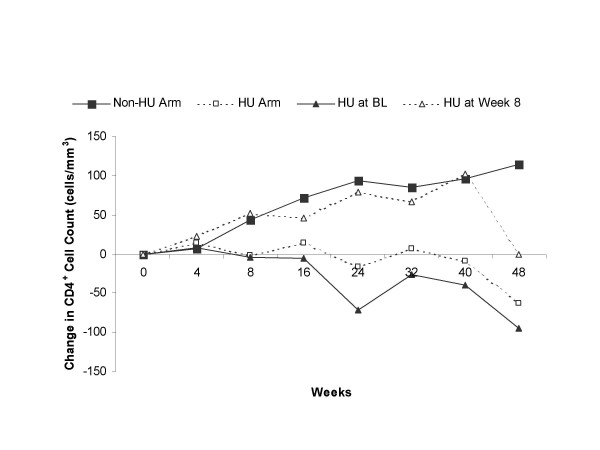
Median change from baseline in CD4+ cell counts in non-HU arm, total HU arm, and HU arm that began HU at baseline and at week 8. BL = baseline; HU = hydroxyurea.

CD4+ cell counts increased steadily and modestly over 48 weeks in the non-HU arm, whereas they decreased slightly in the HU arm as a whole, fell markedly below baseline in subjects who started HU at the beginning of the study, and increased modestly between Weeks 8 and 40 in subjects who started HU at study week 8 (Fig. 5) Median change from baseline in CD4+ cell count in the non-HU and HU arms was +94 cells/mm^3 ^and -17 cells/mm^3 ^(*P *= 0.028), respectively, at week 24, and +114 cells/mm^3 ^and -63 cells/mm^3 ^(*P *= 0.007), respectively, at week 48. At week 48, the median CD4+ cell count was 403 cells/mm^3 ^in the non-HU arm and 249 cells/mm^3 ^in the HU arm (*P *= 0.168). The proportion of subjects achieving at least a 50-cell increase above baseline in CD4+ cell counts was greater in the non-HU arm at week 24 (55% vs 24%, *P *= 0.092) and week 48 (73% vs 18%, *P *= 0.030).

### Safety

The number of subjects experiencing adverse events was similar in both treatment arms. Grade 1–4 drug-related adverse events reported by ≥ 5% of subjects are presented in Table 2. The majority of clinical adverse events were gastrointestinal or neurological in nature and mild to moderate in intensity. Gastrointestinal discomfort/pain, nausea, nausea/vomiting, headache, neuropathy, dizziness, and malaise/fatigue were reported more frequently in the HU arm. The incidence of treatment-limiting adverse events was also higher in the HU arm than the non-HU arm (17 [57%] vs 5 [21%]. Events that occurred once included nausea and vomiting (4 subjects), nausea (3), diarrhea (2), malaise/fatigue (2), dizziness (2), headache (2), allergic reaction (2), and skin rashes (2) in the HU arm, and nausea (1), diarrhea (1), allergic reaction to medicinal substance (1), disorders of lipid metabolism (1), and depressive disorders (1) in the non-HU arm (some subjects experienced more than 1 adverse event). Serious adverse events in the non-HU and HU arms included possible ABC-related hypersensitivity reactions (1 and 3, respectively), appendicitis (1 each), diarrhea (1 in HU arm), bronchitis (1 in HU arm), cognitive function disorders (1 in HU arm), pneumonia (1 in non-HU arm), and asphyxia resulting in death (1 in non-HU arm; not considered related to study drugs). There were no differences between the non-HU and HU arms regarding the incidence of Grade 3 or 4 laboratory toxicities, which included abnormalities in triglycerides (3 and 2 subjects, respectively), amylase (1 and 2), creatine kinase (2 and 1), alanine aminotransferase (0 and 1), and glucose (1 and 0). No significant hematologic toxicities were observed. The HU arm had only slightly lower total lymphocyte counts (mean ± SD 27.7 ± 7.6 cells/μL vs 30.3 ± 10.3 cells/μL) and white blood cell counts (5.4 ± 1.7 cells/μL vs 5.9 ± 1.6 cells/μL) than the non-HU arm.

**Table 2:** Drug-related adverse events reported by ≥ 5% of subjects

Event by body system	ABC/EFV/ddI (*N *= 24)	ABC/EFV/ddI/HU (*N *= 30)
	*n *(%)	*n *(%)
Ear, nose, and throat		
Nasal signs and symptoms	2 (8)	0
		
Endocrine and metabolic		
Lack of appetite	1 (4)	3 (10)
Lipid metabolism disorders	2 (8)	2 (7)
Weight problems	0	2 (7)
		
Gastrointestinal		
Abdominal distension	1 (4)	2 (7)
Diarrhea	5 (21)	6 (20)
Gaseous symptoms	0	2 (7)
GI discomfort and pain	0	3 (10)
Nausea	3 (13)	9 (30)
Nausea and vomiting	1 (4)	6 (20)
		
Musculoskeletal		
Arthralgia	1 (4)	2 (7)
		
Neurology		
Abnormal dreams	5 (21)	4 (13)
Cognitive function	2 (8)	1 (3)
disorders	2 (8)	1 (3)
Dizziness	1 (4)	4 (13)
Headache	1 (4)	5 (17)
Memory effects	0	2 (7)
Neuropathy	1 (4)	4 (13)
Sleep disorders	0	2 (7)
		
Non-site specific		
ABC hypersensitivity	1 (4)	3 (10)
Malaise and fatigue	2 (8)	6 (20)
		
Psychiatric		
Depressive disorders	2 (8)	1 (3)
		
Skin		
Nail disorders	0	2 (7)
Skin rashes	3 (13)	3 (10)

*Note: *ABC, abacavir; ddI, didanosine; EFV, efavirenz; HU, hydroxyurea

## Discussion

In this prospective proof-of-concept clinical trial, combination therapy with ABC/EFV/ddI maintained modest suppression of HIV-1 RNA levels and increases in CD4+ cell counts through 48 weeks of therapy in a significant proportion of subjects who previously failed to respond to their initial NRTI/PI-containing antiretroviral regimens. Thus, this study showed that changing from a PI-based regimen to an NNRTI-based regimen for virologic failure is associated with a favorable outcome in some patients. While cross-study comparisons are limited by differences in subject populations, comparisons of the virologic findings of NZTA4008 with those from studies in subjects with similar baseline HIV disease characteristics and antiretroviral experience can suggest the relative therapeutic usefulness of ABC/EFV/ddI in this subject population. Thus, ABC/EFV/ddI resulted in more subjects achieving undetectable HIV-1 RNA levels and a more pronounced CD4+ cell count increase at 48 weeks than has been reported with d4T/ABC/EFV/ddI in antiretroviral-experienced subjects who had failed on PI-containing HAART [24].

The addition of HU to the triple regimen did not enhance virologic suppression with ABC/EFV/ddI over the entire 48-week study period, although in the ITT: observed analysis (but not the ITT: M = F analysis), a tendency for greater suppression in the HU group was observed at 24 weeks (HIV-1 RNA <400 copies/mL: 89% vs 67% [non-HU]; <50 copies/mL: 74% vs 57%; *P *>0.05). This trend reversed by week 48, with a slightly higher proportion of subjects in the non-HU arm achieving undetectable HIV-1 RNA at that time according to both assays. In contrast, Lafeuillade et al [24] found that when HU 500 mg twice daily was added to an ABC/EFV/ddI/d4T regimen, the significantly enhanced virologic suppression over ABC/EFV/ddI/d4T alone observed at week 24 was maintained at week 48. As HU selectively depletes a greater number of purine, rather than pyrimidine, nucleotides *in vitro *[10], theoretically greater inhibition of HIV-1 would be expected with purine analog reverse transcriptase inhibitors, such as ddI [25,26]. Where the effect of HU on HAART in antiretroviral-experienced subjects has been compared to the effect in antiretroviral-naïve subjects, a greater HU-potentiating effect on virologic suppression was seen in the -experienced group [21]. It is noteworthy that not all studies of antiretroviral-experienced subjects have shown even short-term enhancement of virologic suppression when HU is added to HAART regimens. Indeed, no change in virologic response was observed by Gonzalez et al [27] in their case-control study in subjects receiving HU-containing HAART (HU dose: 500 mg twice daily) (n = 59) versus non-HU-containing HAART (n = 57) over a median of 18 weeks.

Subsequent to our study, Lori et al [28] showed in RIGHT702 that HU, administered at the low dosage of 600 mg daily (lower than that in our study and most other clinical trials), had a better efficacy and safety profile than that seen with higher dosages. RIGHT702 was a randomized, controlled clinical trial in 115 HIV-infected patients comparing the efficacy and safety of HU at three different daily doses (600, 800–900, or 1200 mg/day) given as once-daily, twice-daily, or three-times-daily regimens with ddI and d4T. A pairwise comparison demonstrated a significantly greater proportion of patients on 600 mg daily than 800–900 mg daily attaining HIV-1 RNA <400 copies/mL at week 24 (primary endpoint) (*P *= 0.027) and week 48 (*P *= 0.03), and HIV-1 RNA <50 copies/mL at week 24 (*P *= 0.013) and week 48 (*P *= 0.028). HIV-1 RNA area under the plasma concentration-time curve (AUC) at week 24 (*P *= 0.016) and week 48 (*P *= 0.001) was also lower in the 600 mg daily groups. The twice-daily dosing interval groups were superior to the once-daily group for all virologic endpoints; however, for the CD4+ count there was a tendency favoring the once-daily dosing. The most efficacious combination of total daily dose and dosing interval for the primary endpoint was HU 300 mg twice daily (*P *= 0.017). The total daily dose groups and the dosing interval groups were quite comparable with respect to adverse events. However, one case of lethal pancreatitis occurred in the HU 1,200 mg/day group.

Our study evaluated the effect on CD4+ count of delayed HU treatment (until 8 weeks post-baseline) compared to HU treatment initiated at the start of the study. HU had a cytopenic effect on the CD4+ cell count and blunted the CD4+ response to ABC/EFV/ddI. This effect was diminished if the addition of HU was delayed from baseline until week 8. Rutschmann et al [22] previously compared the effect of immediate versus delayed (by 12 weeks) addition of HU 500 mg twice daily to a HAART regimen (ddI plus d4T), but they did not assess comparative CD4+ cell effects. The cytopenic effect of HU on CD4+ cell counts has been well documented in other studies of HU, especially those in which ddI was given concurrently [29]. However, a few studies have reported little or no reduction in CD4+ cell counts, or even increases [30-32]. The cytopenic effect appears to be a dose-related rather than a duration-related phenomenon [26]. A strategy of delaying HU administration until several weeks after initiation of a new HAART regimen may have potential value in the treatment of HIV-infected subjects, especially those with low CD4+ cell counts pre-treatment.

ABC/EFV/ddI was generally well tolerated, with the primary adverse events being GI in nature. The addition of HU to this regimen did not affect the incidence of rashes, depressive disorders, diarrhea, cognitive function disorders, or possible ABC-related hypersensitivity reactions, although its addition did increase the incidence of nausea, nausea/vomiting, lack of appetite, headache, dizziness, neuropathy, and malaise/fatigue. Other studies have shown that HU at a dose of ≥500 mg twice daily produces adverse GI events that may be additive to those associated with other concurrently administered drugs [26]. In combination with ddI, the incidence of peripheral neuropathy can also be expected to rise [33]. The addition of HU 500 mg twice daily decreased the tolerability of the regimen, with a higher proportion of subjects discontinuing due to treatment-limiting toxicities. Other studies in which subjects received HU 1000 mg/day have similarly reported a high dropout rate due to adverse events [34,35]. Thus, Biron et al [35] found that approximately one-quarter of subjects receiving HU/ddI-containing antiretroviral therapy discontinued treatment within 12 months. However, unlike in our study, early withdrawal in these other studies was due primarily to hematologic toxicity (pancytopenia, neutropenia, anemia), elevations in amylase or liver function tests, or pancreatitis. In contrast to other long-term studies that evaluated HU in combination with ddI and d4T, we did not observe a greater incidence of hematologic toxicities in the HU arm compared with the non-HU arm. This may be due in part to the relative lack of myelosuppression with ABC and EFV [36,37].

This study had several limitations in that it comprised a small sample size (especially at week 48: 10 and 11 in the HU and non-HU arms, respectively), included participants with relatively low baseline HIV-1 RNA values, involved differing numbers of subjects in the treatment arms, and had a high withdrawal rate. HU was randomized against a combination that was highly suppressive, and this could be viewed as a formidable situation in which to verify increased efficacy. As HU was added to the 3-drug combination regimen rather than substituted for one of the regimen components, the occurrence of additional adverse events in the HU arm compared to the non-HU arm is not surprising. The use of non-enteric-coated ddI in our study may have been responsible for greater safety concerns than would have been the case had an enteric-coated ddI formulation been given. Indeed, in an *in vitro *study, Foli et al [38] showed that HU increases mitochondrial toxicity when given with high doses of ddI. Once-daily non-enteric-coated formulation of ddI (which was used in the present study) results in higher maximal blood concentrations compared to the same doses of enteric-coated ddI, thus making mitochondrial toxicity more likely. Our study was also limited because it was not powered to show significant differences between subjects who received HU versus those who did not.

## Conclusion

In conclusion, the results of this study showed that in subjects who have failed initial NRTI/PI-containing regimens, ABC/EFV/ddI may result in modest virologic suppression and increases in CD4+ cell counts. Although no additional enhancement of virologic response was seen over 48 weeks when HU was given concurrently with ABC/EFV/ddI, the above mentioned limitations of this study make it difficult to make generalizable conclusions about the ultimate value of HU in a HAART regimen. Recent data reported for HU and ddI suggest that future efficacy/safety studies of HU in HAART regimens should evaluate an HU dosage no greater than 600 mg daily and use an enteric-coated rather than a non-enteric-coated formulation of ddI if the latter drug is to be administered concurrently.

## Competing interests

The author(s) declare that they have no competing interests.

## Authors' contributions

JEH, QL, BFP, and JWS conceived the study design. SS, CJC, DSB, and KTT reviewed and approved the study design. QL provided the statistical methods for the study and performed the statistical analysis of the results. JEH, QL, BFP, and JWS wrote, reviewed and edited the protocol. GEP drafted the manuscript and evaluated hydroxyurea data previously published in antiretroviral studies described in Background and Discussion. SS, CJC, DSB, KTT, JEH, QL, BFP, and JWS reviewed and edited the manuscript. SS, CJC, DSB, and KTT enrolled study subjects. BFP, JWS, and JEH monitored the study. JEH, QL, BFP, and JWS evaluated the clinical data from the study. BFP set up the study at study sites. JEH contributed to secure funding. All authors read and approved the final manuscript.

## Pre-publication history

The pre-publication history for this paper can be accessed here:


